# Association of Uric Acid in Serum and Urine with Arterial Stiffness: Hanzhong Adolescent Hypertension Study

**DOI:** 10.1155/2020/1638515

**Published:** 2020-07-16

**Authors:** Yang Wang, Xiao-Yu Zhang, Wei-Hua Gao, Ming-Fei Du, Chao Chu, Dan Wang, Chen Chen, Yue Yuan, Qiong Ma, Yue-Yuan Liao, Yu Yan, Ke-Ke Wang, Jie Zhang, Ke Gao, Chun-Hua Li, Hao Li, Qiong Ma, Jia-Wen Hu, Ting Zou, Yue Sun, Min Li, Hao-Wei Zhou, Hao Jia, Jian-Jun Mu

**Affiliations:** ^1^Department of Cardiovascular Medicine, First Affiliated Hospital of Xi'an Jiaotong University, 277 Yanta West Road, 710061 Xi'an, China; ^2^Key Laboratory of Molecular Cardiology of Shaanxi Province, 277 Yanta West Road, 710061 Xi'an, China; ^3^Department of Cardiology, Xi'an No.1 Hospital, 30 Fan Alley, South Street, 710002 Xi'an, China; ^4^Department of Cardiology, Xi'an No.4 Hospital, 21 Jiefang Road, 710004 Xi'an, China; ^5^Department of Ophthalmology, Xi'an No.4 Hospital, 21 Jiefang Road, 710004 Xi'an, China; ^6^Department of Critical Care Medicine, First Affiliated Hospital of Xi'an Jiaotong University, 277 Yanta West Road, Xi'an 710061, China; ^7^Department of Stomatology, First Affiliated Hospital of Xi'an Jiaotong University, 277 Yanta West Road, Xi'an 710061, China; ^8^Department of Cardiovascular Surgery, First Affiliated Hospital of Xi'an Jiaotong University, 277 Yanta West Road, Xi'an 710061, China

## Abstract

**Background:**

Hyperuricemia has long been associated with increased cardiovascular risk, and arterial stiffness is proposed as a mediator. The present study is aimed at examining the associations of uric acid (UA) in blood and urine with arterial stiffness in a Chinese cohort.

**Methods:**

A total of 2296 participants (mean age: 43.0 years) from our previously established cohort of Hanzhong Adolescent Hypertension Study were included. The participants were classified as subjects with or without arterial stiffness, which was defined as brachial-ankle pulse wave velocity (baPWV) ≥ 1400 cm/s and/or carotid intima-media thickness (CIMT) ≥ 0.9 mm. Multivariate regression analyses were used to examine the relationship between serum and urinary UA and the risk of arterial stiffness after adjusting for age, gender, systolic blood pressure, fasting glucose, BMI, heart rate, total cholesterol, and triglycerides.

**Results:**

baPWV was positively correlated with urinary uric acid/creatinine ratio (uUA/Cre) (*β* = 0.061, *P* < 0.001), while CIMT was correlated with uUA/Cre (*β* = 0.085, *P* < 0.001) and fractional excretion of uric acid (FEUA) (*β* = 0.044, *P* = 0.033) in all subjects. In addition, uUA/Cre was significantly associated with the risk of high baPWV [1.032 (1.019-1.045)] and arterial stiffness [1.028 (1.016-1.040)].

**Conclusion:**

Our study showed that urinary UA excretion was significantly associated with the risk of arterial stiffness in Chinese adults. These findings suggest that UA, especially urinary UA, may be used as a simple, noninvasive marker for early detection of arterial stiffness in otherwise healthy subjects.

## 1. Introduction

Arterial stiffness is a strong predictor of cerebrovascular and cardiovascular diseases not only in patients with high risk but also in the general population [1–3]. Several methods for quantifying arterial stiffness have been proposed. The most commonly used technique is measurement of the pulse-wave velocity (PWV), which provides a noninvasive method of assessing stiffness along an arterial section in clinical settings [4, 5]. Brachial-ankle pulse wave velocity (baPWV), which reflects the function of the artery, is a classic artery elasticity measure and an independent predictor for cardiovascular events [4–6]. Carotid intima-media thickness (CIMT) reflects early structural changes in artery walls and is considered a window to systemic vascular arterial stiffness [7, 8]. BaPWV has also been shown to correlate positively with CIMT [9]. BaPWV and CIMT, especially when combined, are useful in predicting future vascular events [10].

Uric acid (UA) is the final oxidation product of purine metabolism. For decades, it has been hypothesized that the antioxidant properties of UA might be protective against oxidative stress, inflammation, and cell injury [11]. Recent studies now suggest that serum uric acid (SUA) is an independent risk factor for cardiovascular and renal disease, particularly in patients with diabetes, hypertension, or heart failure [12]. In addition, although a few studies have investigated the relationship between SUA and arterial stiffness, the results were inconsistent [13–15]. The majority of them investigated primarily on a basic disease, and only baPWV or CIMT was used to represent arterial stiffness. However, there are few reports in a general population regarding these associations.

The kidney plays a key role in regulation of circulating UA levels. Approximately 70% of UA is easily filtered into the renal tubule, and 90% of filtered UA is reabsorbed by the S1 segment of the proximal convoluted tubule. Approximately 10% of filtered UA is finally excreted [16]. Previous evidence demonstrated that elevated urinary UA was a suspected risk factor for calcium oxalate kidney stones in subjects with calcium nephrolithiasis [17, 18]. Recent studies have shown that the urinary UA may be used as a simple, noninvasive marker of the severity of disease and mortality. For example, the urinary uric acid/creatinine ratio (uUA/Cre) has been found to be remarkably higher in hypoxic premature infants or in neonates with birth asphyxia, and this ratio was correlated significantly with the clinical severity of the disease [19–21]. In addition, we recently show that uUA/Cre was significantly associated with the risk of subclinical renal damage (SRD) and its progression in Chinese adults [22]. However, no study has investigated the relationship between urinary UA and arterial stiffness.

In the present study, based on our previously established cohort [23–25], we aimed to investigate the associations of UA in blood and urine with arterial stiffness as measured by increased baPWV and CIMT.

## 2. Materials and Methods

### 2.1. Study Sample

In March and April 1987, we established the cohort of Hanzhong Adolescent Hypertension Study based on a baseline survey of 4623 adolescents aged 6–15 years in over 20 schools of three towns (Qili, Laojun, and Shayan) in Hanzhong, Shaanxi, China. This is an ongoing prospective, population-based cohort study of children and adolescents who regularly undergo follow-ups for investigating the development of cardiovascular risk factors originating in children and young adults. Details of the study protocol have been published elsewhere [23–25].

**Figure 1:**
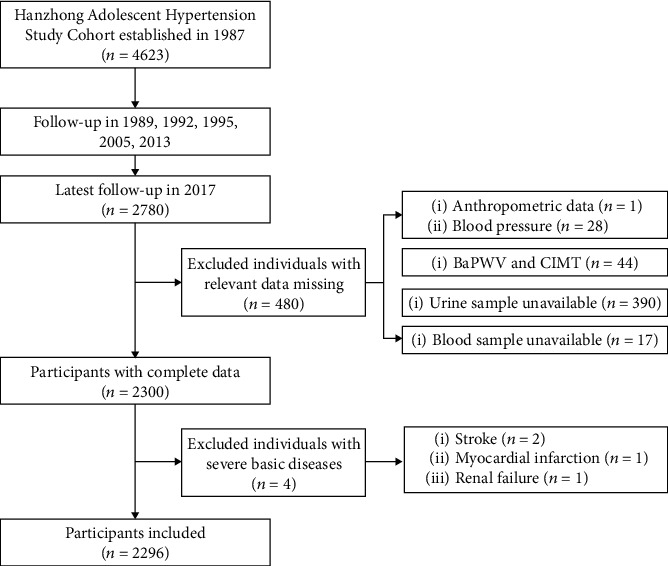
Flow diagram for recruitment of participants.

In this study, we followed up this cohort from April to July 2017, and a total of 2780 were followed up this time. The total rate of this follow-up was 60.3%, which was very rare for such a long-term follow-up. The participant selection process is described in Figure 1. Participants were excluded if they had missing data on serum or urinary biochemistry (*n* = 17 and 390, resp.), baPWV and CIMT (*n* = 44), blood pressure (*n* = 28), weight and height (*n* = 1), and if they had a self-identified history of stroke, coronary heart disease, or renal failure (*n* = 4), leaving 2296 subjects for the primary analyses.

Written informed consent was obtained from all participants, and the Ethics Committee of the First Affiliated Hospital of Xi'an Jiaotong University approved the study protocol (code: 2015–128). The study conducted in according with principles of the Helsinki Declaration (2008). This trial is registered with NCT02734472 (https://www.clinicaltrials.gov).

### 2.2. Anthropometric Measurements

Participants completed standardized questionnaires that inquired about medical history, current medications, alcohol and tobacco use, physical activity, and family history. Body weight and height were measured. Body mass index (BMI) was calculated as weight (kg) divided by height (m^2^). Participants who reported continuous or cumulative smoking for 6 months or more were defined as cigarette smokers [26]. Alcohol consumption was defined as subjects who claimed that they consumed alcohol (liquor, beer, or wine) every day and that consumption lasted for 6 months [27]. Blood pressure (BP) was measured in the sitting position using a standard mercury sphygmomanometer as previously described [28–31]. Hypertension was defined as a systolic BP of ≥140 mm Hg, a diastolic BP ≥ 90 mm Hg, or as the use of antihypertensive agents according to subjects' self-report or pharmacy data.

### 2.3. Laboratory Investigations

All subjects were instructed to avoid alcohol, animal offal, seafood, coffee/tea, beans, and heavy physical activity prior to their sample collection a week ago. Venous blood samples were obtained from all participants after overnight fasting for the determination of serum uric acid (SUA), serum creatinine, serum glucose, and lipid profile (total cholesterol, triglycerides, low-density lipoprotein (LDL), and high-density lipoprotein (HDL)), according to established methods [23, 25, 31].

The first-void and mid-stream urine was collected, followed by venous blood sampling. Urinary concentrations of UA, creatinine, and albumin were measured by an automatic biochemical analyser (Hitachi, Tokyo, Japan). Details of these assays were described previously [22, 23, 31]. UA is transported in the proximal tubule by secretory and reabsorbing transporters, and its handling is a useful marker of proximal tubular function [32, 33]. Fractional excretion of uric acid (FEUA) can be calculated after a simultaneous collection of blood and urine using the standard formula: ([urine uric acid] × [serum creatinine])/([urine creatinine] × [serum uric acid]) × 100, expressed as percentage. eGFR was calculated using the formula adapted from the Modification of Diet in Renal Disease equation on the basis of data from Chinese subjects with CKD [34]. The specific formula is as follows: eGFR = 175 × serum creatinine^−1.234^ × age^−0.179^(×0.79 for women), where serum creatinine concentration is in milligrams per deciliter and age is in years.

### 2.4. Markers of Arterial Stiffness

BaPWV was measured using a volume-plethysmographic device (BP-203RPEII, Nihon Colin, Japan) as previously reported [25, 30, 35–37]. The average value of baPWV values on both sides was used for analysis. BaPWV ≥ 1400 cm/s is an independent variable for risk stratification by Framingham score and for discrimination of subjects with cardiovascular disease. Therefore, high baPWV was defined as baPWV ≥ 1,400 cm/s [30, 36].

The CIMT, defined as the distance the distance from the intima-luminal interface to the media-adventitial interface, was measured as described elsewhere [25, 38]. CIMT was bilaterally visualized and measured at the plaque-free area of the common carotid arteries (10–20mm proximal to the tip of the flow divider). Three images were obtained for both common carotid arteries, and the average of the six measured was used for analysis. The same sonographer who was blinded to the subjects' clinical status carried out all the measurements. According to 2018 Chinese guidelines for the management of hypertension, individuals with early stages of arterial stiffness were defined as those with a nonatherosclerotic CIMT ≥ 0.9 mm [12]. In the current study, we defined arterial stiffness as baPWV ≥ 1400 cm/s and/or CIMT ≥ 0.9 mm as previously reported [25, 38].

### 2.5. Statistical Analysis

Data are expressed as the means ± standard deviations for normally distributed values, as medians (inter-quartile range) for nonnormally distributed values, and as percentages. Significant differences between the groups were calculated using Student's *t*-test, the Mann–Whitney test, and *χ*^2^ test as appropriate. The partial correlation coefficient *R* was measured to assess the relationship between two variables. We performed regression analyses to test associations of UA in serum and urine with arterial stiffness with linear and logistic regression analyses. These analyses were multivariate, adjusting for traditional cardiovascular risk factors and potential confounders. All statistical analyses were conducted using SPSS 16.0 (SPSS, Inc., Chicago, IL). *P* < 0.05 was considered statistically significant.

## 3. Results

### 3.1. Characteristics of Participants

Table 1 presents the characteristics of all subjects according to arterial stiffness status. The prevalence of men, alcohol drinking, smoking, diabetes, and hypertension and age, SBP, DBP, total cholesterol, blood glucose, triglycerides, LDL, SUA, serum creatinine, baPWV, and CIMT were higher in participants with arterial stiffness than in those without arterial stiffness, but HDL-C and FEUA were higher in those without arterial stiffness.

### 3.2. Correlations of Serum and Urinary UA Levels with baPWV and CIMT

**Figure 2:**
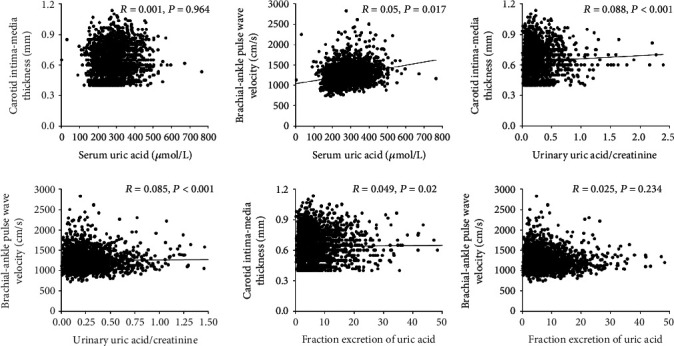
Correlations among baPWV and CIMT with SUA, uUA/Cre and FEUA in all subjects. Partial correlation analyses were used after adjusting for age, gender, hypertension, diabetes, BMI, heart rate, total cholesterol, and triglycerides.

Partial correlation analyses showed that baPWV was positively correlated with SUA (*r* = 0.05, *P* = 0.017) and uUA/Cre (*r* = 0.085, *P* < 0.001, Figure 2). CIMT was correlated with uUA/Cre (*r* = 0.088, *P* < 0.001) and FEUA (*r* = 0.049, *P* = 0.02) (Figure 2).

Multiple linear regression analysis found that baPWV was closely correlated with age, BMI, SBP, fasting glucose, heart rate, total cholesterol, SUA, and uUA/Cre (*β* = 0.061, *P* < 0.001), while it was inversely correlated with sex (*R*^2^ = 0.463). In addition, CIMT was positively correlated with age, BMI, fasting glucose, total cholesterol, uUA/Cre (*β* = 0.085, *P* < 0.001), and FEUA (*β* = 0.044, *P* = 0.033) but negatively correlated with sex in all subjects (*R*^2^ = 0.047) (Table 2).

### 3.3. Associations of Serum and Urinary UA Levels with Arterial Stiffness

To extend this finding to clinical practice, we further investigated the association between UA and high baPWV (baPWV ≥ 1400 cm/s) or increased CIMT (CIMT ≥ 0.9 mm) based on multiple logistic regressions. uUA/Cre was significantly associated with the risk of high baPWV [1.032 (1.019-1.045), *P* < 0.001] but not increased CIMT [1.546 (0.680-3.515), *P* = 0.299] after adjusting for confounding factors. However, no significant association was found in either SUA or FEUA (Table 3).

To further comprehensively and accurately evaluate arterial stiffness, we combined CIMT and baPWV and defined arterial stiffness as baPWV ≥ 1400 cm/s and/or CIMT ≥ 0.9 mm. We found that after adjusting for multiple confounders, uUA/Cre [1.028 (1.016-1.040)] was significantly associated with the risk of arterial stiffness (Table 4). SUA (*P* = 0.268) and FEUA (*P* = 0.719) did not remain in the final model.

### 3.4. Sensitivity Analysis

To further exclude the potential influence of medication use, including antihypertensive drugs, we removed subjects with hypertension, diabetes, or hyperuricemia under treatment (*n* = 91). As shown in Supplemental Tables 1-2, all the results remained similar after adjustment for potential confounders.

## 4. Discussion

To the best of our knowledge, the present study is the first to evaluate the relationship between urinary UA excretion and the risk of arterial stiffness. Interestingly, we showed that uUA/Cre was significantly associated with baPWV and CIMT. In addition, when baPWV and CIMT are combined to comprehensively evaluate arterial stiffness, we found that uUA/Cre is significantly associated with an increased risk of arterial stiffness. These data indicate that the urinary UA may act as a simple noninvasive, cost-effective, single biochemical marker for assessing the severity of arterial stiffness.

Uric acid homeostasis is determined by the balance between production, intestinal secretion, and renal excretion [39]. The renal proximal tubule is responsible for almost all renal urate transport and is the primary site of urate reabsorption [32]. Several previous studies indicated that urinary UA may be associated with the disease incidence and development. Li et al. [40] reported that urinary UA was negatively associated with albuminuria in a cross-sectional study of 200 Chinese patients with chronic kidney disease. Ozanturk et al. [41] revealed significant correlation of nocturnal hypoxemia with UA excretion and uUA/Cre in a group of patients including obstructive sleep apnea (OSA) and chronic obstructive pulmonary disease (COPD) patients. Moreover, recent studies suggest that uUA/Cre is simple, noninvasive, painless, and economical in investigation for the diagnosis of perinatal asphyxia. For example, Chen et al. [42] showed that the uUA/Cre was remarkably higher in hypoxic premature infants than in hypoxic-term infants. Bader et al. [19] reported that uUA/Cre was elevated in infants with perinatal asphyxia compared with the control group, and that this ratio was correlated significantly with the clinical severity of the disease. Nariman et al. [43] found that uUA/Cre increased with the severity of disease and was associated with longer duration of stay and adverse outcome in all NICU-admitted neonates. The essence of neonatal asphyxia is hypoxia. Hypoxia can lead to energy metabolism disorders, excessive production of oxygen free radicals, accumulation of adenosine diphosphate (ADP), and adenosine monophosphate (AMP) caused by incompletely oxidization, which makes excessive adenosine, inosine, and hypoxanthine. After these substances are catabolized, uric acid in the blood increases, which leads to increased excretion of uric acid in the urine [44]. Therefore, the detection of UA in urine may help measure the degree of oxidative damage. To the best of our knowledge, this is the first study to investigate the relationship between urinary UA and baPWV, and we showed that urinary excretion of UA was significantly associated with baPWV and a risk of high baPWV in the general population. Cao and Wang [45] indicated that TAS and arterial elasticity are decreased while arterial stiffness is increased in elderly hypertensive patients. The decline in antioxidant capacity may be responsible for vascular damage and arterial elasticity decrease in elderly hypertensive patients. Therefore, we assume that oxidative damage may be the bridge between uric acid excretion and arterial stiffness.

Subclinical arterial stiffening may already exist in healthy individuals, and a high UA level may be useful in predicting the incidence of arterial stiffening. Many studies have investigated the relationship between SUA and arterial stiffness in various populations, with inconsistent findings [13–15]. However, majority of the studies have been conducted on patients with various disorders, such as hypertension, diabetes, and CKD. Moreover, only baPWV or CIMT was used to evaluate arterial stiffness in previous studies [13–15]. BaPWV can be evaluated as an arterial functional distensibility, whereas CIMT is considered to be a parameter of arterial structural change. In fact, structural and functional alterations of the arteries can suggest a functional impairment long before the appearance of clinical lesions. Nagai et al. [10] reported that baPWV and CIMT, especially when combined, are useful in predicting future vascular events. In the present study, when baPWV and CIMT are combined, we failed to show any association between SUA and arterial stiffness. Interestingly, our data show that urinary excretion of UA was significantly associated with the risk of arterial stiffness in the general population. Very recently, we also showed that urinary UA excretion was significantly associated with the risk of subclinical renal damage, defined as slightly increased albuminuria or decreased eGFR in Chinese adults [22]. In fact, UA per se can be detrimental to the kidneys, as shown in basic studies. UA can induce vascular smooth muscle cell proliferation, chronic inflammation, endothelial dysfunction, and activation of the renin angiotensin system (RAS) in the kidney, which may be possible mechanisms for renal damage [46, 47].

Some limitations of our study merit consideration. First, our results were obtained from northern-Chinese individuals and consequently may not be generalizable to other ethnic groups with different demographics. In addition, all subjects in this study were youth and middle aged between 36 and 45 years during the follow-up at 2017, and thus, the findings may not be generalizable to other age groups. Finally, a single measurement may be insufficient to assess the urinary UA excretion of individuals. Urinary UA excretions may change according to the urine sampling time because UA excretion may be influenced by the time at which food is consumed or the drugs they take. However, all subjects were instructed to avoid alcohol, animal offal, seafood, coffee/tea, beans, and heavy physical activity prior to their sample collection a week ago. Also, participants under medication were excluded to minimize the confounders in our sensitivity analysis, and the results were similar.

In summary, our study shows that urinary UA excretion was significantly associated with the risk of arterial stiffness in Chinese adults. However, we failed to find a significant relationship between serum UA and arterial stiffness. These findings suggest that UA, especially urinary UA, may be used as a simple, noninvasive marker for early detection of arterial stiffness in otherwise healthy subjects. Future studies are necessary to confirm the relationship between urinary UA and subclinical arterial stiffness and elucidate the potential mechanisms underlying this association.

## Figures and Tables

**Table 1:** Characteristics of participants categorized by arterial stiffness (AS) status.

Characteristics	All	Subjects with AS	Subjects without AS	*P* value
No. of subjects (%)	2296 (100.0)	581 (25.3)	1715 (74.7)	—
Gender (male, %)	55.3	73.3	49.2	<0.001
Age (years)	43.0 (40.0-45.0)	43.0 (40.0-45.0)	43.0 (40.0-45.0)	<0.001
Alcohol consumption (%)	29.2	37.3	26.5	<0.001
Current smoking (%)	43.0	55.4	38.8	<0.001
Diabetes mellitus (%)	4.0	7.4	2.9	<0.001
Hypertension (%)	20.6	50.3	10.5	<0.001
BMI (kg/m^2^)	23.8 (21.9-26.0)	24.7 (22.7-26.8)	23.5 (21.6-25.6)	0.368
Heart rate (beats/min)	69.0 (63.5-75.5)	71.5 (65.8-78.3)	68.5 (63.0-74.5)	<0.001
SBP (mmHg)	121.3 (112.-131.3)	133.3 (124.3-146.7)	110.0 (118.0-126.3)	<0.001
DBP (mmHg)	76.0 (69.0-84.0)	86.0 (78.7-93.3)	73.7 (67.7-79.7)	<0.001
SUA (*μ*mol/L)	279.2 (225.0-335.3)	305.8 (263.0-360.1)	269.8 (218.6-324.2)	<0.001
Fasting glucose (mmol/L)	4.57 (4.28-4.91)	4.67 (4.34-5.01)	4.54 (4.26-4.86)	<0.001
Total cholesterol (mmol/L)	4.51 (4.04-5.01)	4.64 (4.17-5.15)	4.47 (4.01-4.97)	<0.001
Triglycerides (mmol/L)	1.33 (0.96-1.95)	1.55 (1.01-2.23)	1.26 (0.92-1.84)	<0.001
LDL (mmol/L)	2.50 (2.13-2.91)	2.58 (2.22-3.01)	2.46 (2.10-2.86)	<0.001
HDL (mmol/L)	1.15 (0.99-1.33)	1.10 (0.97-1.28)	1.16 (1.00-1.35)	<0.001
Serum creatinine (*μ*mol/L)	76.7 ± 14.2	79.6 ± 14.8	75.7 ± 13.9	<0.001
eGFR (mL/min/1.73 m^2^)	97.2 (86.9-110.0)	95.6 (85.9-108.7)	97.6 (87.2-110.3)	0.150
uUA/Cre	0.20 (0.12-0.33)	0.20 (0.11-0.32)	0.20 (0.12-0.33)	0.566
FEUA	5.19 (3.04-9.17)	5.07 (2.76-8.58)	5.44 (3.13-9.37)	0.025
CIMT (mm)	0.62 (0.53-0.75)	0.68 (0.57-0.83)	0.60 (0.53-0.70)	<0.001
baPWV (cm/s)	1219.0 (1093.6-1374.1)	1498.0 (1428.0-1624.8)	1163.5 (1061.5-1265.0)	<0.001

AS: arterial stiffness; BMI: body mass index; SBP: systolic blood pressure; DBP: diastolic blood pressure; SUA: serum uric acid; LDL: low-density lipoprotein; HDL: high-density lipoprotein; uUA/Cre: urinary uric acid/creatinine ratio; FEUA: fraction excretion of uric acid; baPWV: brachial-ankle pulse wave velocity; CIMT: carotid intima-media thickness. Nonnormally distributed variables are expressed as the median (interquartile range). All other values are expressed as mean ± SD or *n*, %.

**Table 2:** Relationship between various characteristics and baPWV and CIMT.

Characteristics	baPWV		CIMT	
	*β*	*P* value	*β*	*P* value
Gender (male)	-0.119	<0.001	-0.135	<0.001
Age (years)	0.102	<0.001	0.082	<0.001
BMI (kg/m^2^)	-0.073	<0.001	0.094	<0.001
SBP (mmHg)	0.596	<0.001	0.005	0.828
Fasting glucose (mmol/L)	0.044	0.006	0.059	0.006
Heart rate (beats/min)	0.092	<0.001	0.024	0.261
Total cholesterol (mmol/L)	0.039	0.014	0.046	0.032
Triglycerides (mmol/L)	0.003	0.846	-0.027	0.238
SUA (*μ*mol/L)	0.056	0.005	0.021	0.422
uUA/Cre	0.061	<0.001	0.085	<0.001
FEUA	0.014	0.370	0.044	0.033

BaPWV: brachial-ankle pulse wave velocity; CIMT: carotid intima-media thickness; BMI: body mass index; SBP: systolic blood pressure; SUA: serum uric acid; uUA/Cre: urinary uric acid/creatinine ratio; FEUA: fraction excretion of uric acid.

**Table 3:** Association between various characteristics and the risk of high baPWV or increased CIMT.

Characteristics	High baPWV (≥1400 cm/s)		Increased CIMT (≥0.9 mm)	
	Odds ratios (CI)	*P* value	Odds ratios (CI)	*P* value
Gender (male)	0.458 (0.334-0.629)	<0.001	0.405 (0.240-0.684)	0.001
Age (years)	1.063 (1.025-1.103)	0.001	1.137 (1.078-1.198)	<0.001
BMI (kg/m^2^)	0.938 (0.898-0.979)	0.004	1.074 (1.004-1.150)	0.038
SBP (mmHg)	1.097 (1.085-1.108)	<0.001	1.005 (0.992-1.017)	0.470
Fasting glucose (mmol/L)	1.018 (0.922-1.125)	0.721	1.131 (1.000-1.280)	0.050
Heart rate (beats/min)	0.997 (0.922-1.125)	0.746	0.997 (0.976-1.018)	0.746
Total cholesterol (mmol/L)	1.202 (1.028-1.405)	0.021	1.135 (0.883-1.458)	0.322
Triglycerides (mmol/L)	1.007 (0.918-1.105)	0.877	0.897 (0.742-1.084)	0.260
SUA (*μ*mol/L)	1.001 (0.999-1.003)	0.165	0.999 (0.996-1.002)	0.680
FEUA	0.997 (0.987-1.008)	0.624	1.001 (0.990-1.013)	0.820
uUA/Cre	1.032 (1.019-1.045)	<0.001	1.546 (0.680-3.515)	0.299

Logistic regression analyses were used to test the risk of high baPWV and increased CIMT, after adjustment for age, gender, SBP, fasting glucose, BMI, heart rate, total cholesterol, and triglycerides. CI: confidence interval; BaPWV: brachial-ankle pulse wave velocity; CIMT: carotid intima-media thickness; BMI; body mass index; SBP: systolic blood pressure; SUA: serum uric acid; FEUA: fraction excretion of uric acid; uUA/Cre: urinary uric acid/creatinine ratio.

**Table 4:** Association between various characteristics and the risk of arterial stiffness.

Characteristics	Odds ratios (CI)	*P* value
Gender (male)	0.451 (0.338-0.601)	<0.001
Age (years)	1.186 (1.049-1.124)	<0.001
BMI (kg/m^2^)	0.964 (0.927-1.003)	0.070
SBP (mmHg)	1.078 (1.069-1.088)	<0.001
Fasting glucose (mmol/L)	1.051 (0.962-1.149)	0.273
Heart rate (beats/min)	1.445 (0.864-2.418)	0.161
Total cholesterol (mmol/L)	1.175 (1.018-1.356)	0.027
Triglycerides (mmol/L)	0.987 (0.904-1.077)	0.765
SUA (*μ*mol/L)	1.001 (0.999-1.003)	0.268
FEUA	0.998 (0.990-1.007)	0.719
uUA/Cre	1.028 (1.016-1.040)	<0.001

Logistic regression analyses were used to test the risk of arterial stiffness, after adjustment for age, gender, SBP, fasting glucose, BMI, heart rate, total cholesterol, and triglycerides. CI: confidence interval; BMI: body mass index; SUA: serum uric acid; FEUA: fraction excretion of uric acid; uUA/Cre: urinary uric acid/creatinine ratio.

## Data Availability

The data used to support the findings of this study are available from the corresponding authors upon request.

## Supplementary Materials

Supplementary Materials Table S1: association between various characteristics and the risk of baPWV and CIMT in subjects without medication use (*n* = 2205). Table S2: association between various characteristics and the risk of arterial stiffness in subjects without medication use (*n* = 2205).
