# Supplementary material for: Association of Uric Acid in Serum and Urine with Arterial Stiffness: Hanzhong Adolescent Hypertension Study
Source: Dis Markers. 2020 Jul 16;2020:1638515. doi: 10.1155/2020/1638515 (PMC7382737; doi:10.1155/2020/1638515)
Supplement: Supplementary Materials — Table S1: association between various characteristics and the risk of baPWV and CIMT in subjects without medication use (n = 2205). Table S2: association between various characteristics and the risk of arterial stiffness in subjects without medication use (n = 2205). [file 1638515.f1.zip › Table S1.doc]

**Table S1.** Association between various characteristics and the risk of baPWV and CIMT in subjects without medication use (n=2205).

| Characteristics | High baPWV( ≥ 1400 cm/s) | | Increased CIMT( ≥ 0.9 mm) | |
| --- | --- | --- | --- | --- |
|  | Odds ratios (CI) | *P* value | Odds ratios (CI) | *P* value |
| Gender (Male) | 0.463 (0.300-0.714) | 0.001 | 0.305 (0.153-0.610) | 0.001 |
| Age (years) | 1.072 (1.031-1.116) | 0.001 | 1.158 (1.091-1.230) | 0.001 |
| BMI (kg/m^2^) | 0.928 (0.883-0.975) | 0.003 | 1.056 (0.977-1.142) | 0.167 |
| SBP (mmHg) | 1.053 (1.035-1.071) | <0.001 | 0.987 (0.962-1.013) | 0.331 |
| Fasting glucose (mmol/L) | 0.924 (0.784-1.088) | 0.340 | 1.191 (0.953-1.490) | 0.125 |
| Heart rate (beats/min) | 1.195 (0.526-2.717) | 0.670 | 0.737 (0.196-2.779) | 0.653 |
| Total cholesterol (mmol/L) | 0.944 (0.566-1.572) | 0.824 | 1.339 (0.626-2.865) | 0.451 |
| Triglycerides (mmol/L) | 1.087 (0.883-1.338) | 0.434 | 0.849 (0.583-1.234) | 0.391 |
| SUA (μmol/L) | 1.001 (0.999-1.004) | 0.390 | 0.999 (0.995-1.003) | 0.578 |
| FEUA | 0.990 (0.927-1.058) | 0.774 | 0.998 (0.966-1.030) | 0.885 |
| uUA/Cre | 1.026 (1.012-1.040) | <0.001 | 1.460 (0.432-4.938) | 0.543 |

Logistic regression analyses were used to test the risk of arterial stiffness, after adjustment for age, gender, SBP, fasting glucose, BMI, heart rate, total cholesterol, triglycerides. CI, confidence interval; BMI, body mass index; SBP, systolic blood pressure; SUA, serum uric acid; FEUA, fraction excretion of uric acid; uUA/Cre, urinary uric acid/creatinine ratio.
