# Supplementary material for: Association of Uric Acid in Serum and Urine with Arterial Stiffness: Hanzhong Adolescent Hypertension Study
Source: Dis Markers. 2020 Jul 16;2020:1638515. doi: 10.1155/2020/1638515 (PMC7382737; doi:10.1155/2020/1638515)
Supplement: Supplementary Materials — Table S1: association between various characteristics and the risk of baPWV and CIMT in subjects without medication use (n = 2205). Table S2: association between various characteristics and the risk of arterial stiffness in subjects without medication use (n = 2205). [file 1638515.f1.zip › Table S2.doc]

**Table S2.** Association between various characteristics and the risk of arterial stiffness in subjects without medication use (n=2205).

| Characteristics | Odds ratios (CI) | *P* value |
| --- | --- | --- |
| Gender (Male) | 0.408 (0.275-0.604) | <0.001 |
| Age (years) | 1.097 (1.057-1.137) | <0.001 |
| BMI (kg/m^2^) | 0.952 (0.911-0.995) | 0.030 |
| SBP (mmHg) | 1.034 (1.019-1.050) | <0.001 |
| Fasting glucose (mmol/L) | 0.975 (0.844-1.126) | 0.732 |
| Heart rate (beats/min) | 1.060 (0.502-2.239) | 0.878 |
| Total cholesterol (mmol/L) | 0.998 (0.632-1.578) | 0.994 |
| Triglycerides (mmol/L) | 1.035 (0.855-1.253) | 0.723 |
| SUA (μmol/L) | 1.001 (0.999-1.003) | 0.499 |
| FEUA | 0.994 (0.960-1.028) | 0.711 |
| uUA/Cre | 1.021(1.009-1.034) | 0.001 |

Logistic regression analyses were used to test the risk of arterial stiffness, after adjustment for age, gender, SBP, fasting glucose, BMI, heart rate, total cholesterol, triglycerides. CI, confidence interval; BMI, body mass index; SBP, systolic blood pressure; SUA, serum uric acid; FEUA, fraction excretion of uric acid; uUA/Cre, urinary uric acid/creatinine ratio.
